# Big Data–Driven Health Portraits for Personalized Management in Noncommunicable Diseases: Scoping Review

**DOI:** 10.2196/72636

**Published:** 2025-06-05

**Authors:** Haoyang Du, Jianing Yu, Dandan Chen, Jingjie Wu, Erxu Xue, Yufeng Zhou, Xiaohua Pan, Jing Shao, Zhihong Ye

**Affiliations:** 1 Sir Run Run Shaw Hospital Hangzhou China; 2 School of Nursing and Institute of Nursing Research, School of Medicine Zhejiang University Hangzhou China; 3 Department of Nursing The Fourth Affiliated Hospital of School of Medicine, and International School of Medicine, International Institutes of Medicine Zhejiang University Yiwu China; 4 Ningbo University Affiliated Hospital Ningbo China; 5 Binjiang Research Institute of Zhejiang University School Zhejiang University Hangzhou China; 6 The D H Chen School of Universal Health Zhejiang University Hangzhou China

**Keywords:** health portraits, big data, non-communicable disease, NCDs, management

## Abstract

**Background:**

Health portraits powered by big data integrate diverse health-related data into actionable insights, thereby facilitating precise risk prediction and personalized management of noncommunicable diseases (NCDs). Despite their promise, the adoption and application of health portraits remain fragmented, primarily due to the lack of a standardized conceptual and methodological framework necessary to fully harness their capabilities.

**Objective:**

This study aimed to systematically map and categorize existing research on health portraits in the context of NCD management, evaluate how big data has been used through the lens of the 3V (volume, velocity, and variety) framework, assess the extent of external validation and comprehensiveness, and identify challenges, emerging opportunities, and future research directions in this field.

**Methods:**

A scoping review was conducted following the PRISMA-ScR (Preferred Reporting Items for Systematic Reviews and Meta-Analyses extension for Scoping Reviews) guidelines and 6-step framework of Levac et al. A comprehensive search was performed in PubMed, Embase, EBSCO, Ovid, Scopus, Web of Science, and Springer Link, focusing on observational and interventional studies using big data, public databases, electronic health record systems, wearables, and sensors for NCD management from January 2014 to July 2024. Data extraction included study characteristics, modeling approaches, and external validation. Analytical synthesis was conducted using keyword analysis, the 3V framework, and visual tools such as scatter plots, heat maps, and radar charts.

**Results:**

A total of 8707 records were identified, and 89 studies were included for full-text analysis. These studies were categorized into 4 types of health portraits: diagnostic, prognostic, monitoring, and recommender. Evaluation based on the 3V framework showed that only 17.78% of studies met all 3 criteria. In terms of volume, structured data were widely used (64.29%-100% depending on portrait type), while unstructured data usage varied significantly (19.05%-93.33%). Regarding velocity, monitoring and recommender portraits showed high reliance on digital interactive data (over 85%). For variety, only 31.11% of studies incorporated all 3 data attributes (natural, domain, and specific attributes). In terms of comprehensiveness, only 30% of studies reported the external validation, and only 10% met both the external validation and 3V criteria, with recommender portraits outperforming the other types.

**Conclusions:**

This study provides a standardized lens through which to evaluate the development and application of health portraits in NCD management. The findings underscore the need for more robust data integration strategies and emphasize the importance of artificial intelligence–enabled approaches. Furthermore, enhancing external validation and addressing ethical and privacy considerations are critical for advancing the implementation of personalized health management solutions.

## Introduction

Noncommunicable diseases (NCDs) account for 77% of the projected global disease burden by 2030, claiming 17 million premature annual deaths and imposing unsustainable pressure on health care systems [1-4]. This threat has accelerated a paradigm shift from reactive treatment to proactive health management [5-7], underscoring the need for integrated care across clinical and community settings [1,8-10] with the emerging emphasis on precision medicine approaches [11,12]. Fueling this transition, digital health technologies—from electronic health records (EHRs) to wearables and remote sensing devices—are generating vast multidimensional data streams, offering unprecedented potential for individualized data-driven interventions and resource optimization [13,14].

Within this data-rich landscape, “health portraits” have emerged as a novel framework to organize and interpret complex health data for precision health management [15]. Originally inspired by Alan Cooper's “User Personas” concept in 1998 and later adapted to health care by Liu et al [15], Cooper et al [16], and Pietilä et al [17], health portraits refer to an integrated, person-centered representation that synthesizes heterogeneous data, including symptoms, medical history, biochemical tests, and lifestyle factors, into a unified profile of an individual’s health status. Previous studies have shown that health portraits are typically composed of 3 layers of attributes: natural (eg, age and gender), domain (eg, behavior and preferences), and specific attributes (eg, physiological health and disease risk) [15]. Operationally, their construction follows a multistage pipeline: data acquisition, reprocessing, label mapping, and contextualization [18], enabling the translation of raw data into meaningful insights for personalized health management [19,20] in risk stratification, telehealth delivery, and personalized interventions [21-27].

In recent years, a growing number of health portraits have leveraged big data resources, including large-scale EHRs, patients’ self-reported data, and wearable outputs, driven by the defining features of big data: volume, velocity, and variety (the “3V” framework) [28-30]**.** High-profile examples include the All of Us Research Program in the United States, which integrates genomic, EHR, and wearable data from over 1 million participants to support inclusive biomedical research and personalized health insights. Similarly, IBM Watson Health applies artificial intelligence (AI)–driven analysis of multimodal data (eg, EHRs, genomics) to optimize oncology decision-making, while Oura Ring generates continuous physiological health portraits for disease risk prediction through real-time tracking of heart rate variability, sleep, and activity [31-34]. These initiatives collectively demonstrate the transformative potential of big data–driven health portraits in advancing real-time, personalized, and context-aware NCD care across clinical and everyday settings [35-37].

While numerous studies have explored the construction and application of health portraits, there has been limited effort to systematically map and categorize their functions or application contexts within NCD management. Some researchers have noted that insufficient processing of data volume, poor integration of diverse data types, and inadequate support for real-time updates continue to constrain the scalability and fidelity of health portraits [38,39]. Likewise, an empirical study found that over 50% of code modules for health analytics failed peer review, largely due to test instability and weak validation [40]. Moreover, the mismatch between datasets and scenarios, such as using inpatient clinical data in community settings, further limits the contextual relevance and broader applicability of health portraits [41].

Despite the growing body of work, no systematic reviews to date have classified the functional types and implementation scenarios of health portraits, assessed their alignment with the big-data “3V” framework (volume, velocity, and variety), or examined their external validation status. To support the theoretical advancement and practical application of big data–driven health portraits, this scoping review aims to (1) categorize health portraits' functionalities and application scenarios in NCD care, (2) evaluate 3V alignment and external validation status, and (3) propose an implementation roadmap addressing current limitations.

## Methods

We performed a scoping review based on the PRISMA-ScR (Preferred Reporting Items for Systematic Reviews and Meta-Analyses extension for Scoping Reviews) statement [42] as shown in Multimedia Appendix 1. We followed the 6-step framework outlined by Levac et al [43], who updated and extended the initial framework developed by Arksey and O’Malley [44].

### Search Strategy

We searched PubMed, Embase, EBSCO, Ovid, Scopus, Web of Science, and Springer Link. Search terms included terms describing the concept (health portrait) in combination with population (NCDs) and context (big data). We also included related terms such as “User-centered Design” and “Telemedicine” as search terms because they may be related to the concept or context. The search was restricted to articles published in English or Chinese from January 2014 to July 2024. Furthermore, we scanned reference lists of included publications and published reviews for additional articles. The full search queries can be found in Multimedia Appendix 2.

### Selection Criteria

Population is limited to patients diagnosed with NCDs defined by the World Health Organization [45]. Moreover, we also included studies on a broader range of NCDs where relevant, like obesity, mental disorders, and geriatric syndromes, to ensure a comprehensive review of health portrait types and the methodologies used [46,47].

Concept (health portrait) is defined in this study as the models or programs to stratify or classify patients under a certain characteristic or condition based on characteristic information and process data labels related to health status [15], for example, specific social determinants, health risk scores, and lifestyle behaviors.

Context (big data) is defined as the extent to which the study’s modeling process aligns with the “3Vs” framework—volume, velocity, and variety. Specifically, a study is considered to meet the criteria if it satisfies all of the following: (1) the inclusion of unstructured data; (2) the use of data sources from digital interactive platforms; and (3) the incorporation of patient data covering natural, domain, and specific attributes.

The types of publications included observational studies and interventional studies. Besides, we excluded studies that used only traditional data collection methods (interviews or scales) for data collection. Studies that used public databases, EHR systems, digital application platforms, and wearables and sensors for data collection are included. Detailed explanations about the concept (health portrait), population (NCDs), and context (big data) can be found in Multimedia Appendix 3. The study screening manual is shown in Table 1.

**Table 1:** Eligibility criteria and their rationale.

Eligibility criteria and variable				Rationale
**Inclusion criteria**				
	Population	Patients diagnosed with NCDs^a^	NCDs are prevalent on a vast scale and impose a significant burden on health care management.	
	Concept	The health portrait models or programs to stratify or classify the health status of patients into digital labels	It can present a more digital and clearer reference method for personalized health management.	
	Context	Research data derived from public databases, electronic health care record systems, digital application platforms, and wearables and sensers	Public databases and EHRs^b^ are the most common ways for data collection, and digital application platforms and wearables and sensers provide multimodal datasets, and the data transfer speed is fast.	
	Others	Peer-reviewed	It has greater credibility because the papers have been reviewed by peer experts in the field.	
	Others	Empirical study design	Empirical studies improve the ability to answer the research questions compared with conceptual commentaries or viewpoints.	
	Others	Published between January 2014 and July 2024	It was not after 2014 when big data and the Internet of Things (IoT) were relatively widespread and used in more studies.	
	Others	English or Chinese language	It is about some practical considerations, given the investigators’ language proficiency.	
**Exclusion criteria**				
	Population	Research on patients with infectious diseases	The prognosis, influencing factors and profiling requirements of infectious diseases are quite different from those of NCDs.	
	Concept	Studies not related to health portrait modeling or population stratification	Studies not relative to our research theme or key research questions can hardly help to identify the scope of health portraits.	
	Context	Research data only derived from traditional questionnaires or qualitative studies	Based on a single cross-sectional study design, the data collection uses traditional scales or interviews to obtain information, which has great limitations in terms of data volume, velocity, and variety.	
	Others	Editorials, commentaries, opinion articles, and reports	These types of sources are not based on original research or data analysis, which does not ensure a focus on evidence-driven insights.	

^a^NCD: noncommunicable disease.

^b^EHR: electronic health record.

### Study Selection

To increase the consistency of study screening among reviewers, reviewer 1 piloted the study screening manual for database search and study selection based on title and abstract information available in the databases. Subsequently, reviewer 2 independently cross-checked the study selection of all articles identified in the database search. Both reviewers then discussed results and amended the screening manual before the data charting step. Subsequently, both reviewers 1 and 2 independently piloted the study screening manual for evaluating the eligibility of 10% of all identified full-text reports using a computer-generated random sequence, along with complete data charting for the included articles. Both reviewers then discussed the results and amended the screening manual. Finally, reviewers 1 and 2 independently completed an assessment of the remaining full-text reports for eligibility, along with data charting for all included reports.

### Quality Assessment

This review included both observational studies and interventional studies. To reconcile the scope review’s mandate for breadth with methodological accountability, we used a dual-tiered screening framework according to some previous research [48,49]: (1) prioritizing journals to leverage rigorous peer-review standards and (2) inclusion of methodologically innovative studies despite their limited overall quality, following thorough internal team deliberations to ensure balanced inclusion. While formal quality appraisal tools were intentionally omitted to preserve conceptual mapping flexibility—an accepted scoping review limitation—we embedded critical safeguards: systematic exclusion of studies with high bias risks (randomization, blinding, and allocation concealment) and study type-specific quality screening aligned with Multimedia Appendix 4. This included external validation thresholds for clinical predictive models, health recommender systems, and interventional studies.

### Data Extraction and Synthesis

Reviewer 1 extracted basic information from the included articles provided by the first author: published year, country, study design, participants, modeling approaches, external validation status, sample size, and data usage. We group studies by the scenes, participants, objectives, and modeling approaches of the health portraits, using Python-extracted keywords in the titles and abstracts, and reviewers conduct secondary integration of keywords. An expert panel and the research team determined the 3V framework based on the concept of big data and the comprehensive capability assessment (Multimedia Appendix 5). All findings are synthesized to identify existing status and knowledge gaps for future research. Key results are summarized using the word cloud analysis to identify different types of health portraits, the heat map to identify the existing status of health portraits that meet the requirements of “3V,” as well as the spider diagrams to perform the comprehensive capability of included records meeting the requirements of both the external validation and “3V.” Any disagreements on study selection and data charting during pilot testing were resolved by consensus, or otherwise with a tiebreaker by reviewer 3 if needed.

## Results

**Figure 1:**
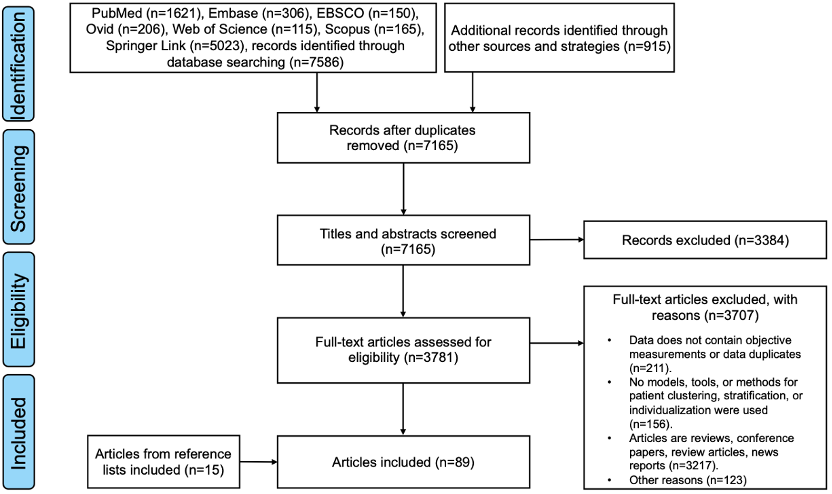
Inclusion flowchart. The 4 phases of article selection follow the PRISMA-ScR (Preferred Reporting Items for Systematic Reviews and Meta-Analyses extension for Scoping Reviews) guidelines.

A total of 7888 articles were searched in 7 databases for the initial and secondary searches, and 915 articles were manually searched. Of the 8707 records, after the removal of duplicates, 7165 (73.8%) records remained, and we screened titles and abstracts. In this screening, 47.2% (3384/7165) of the records were excluded, and the remaining 52.8% (3781/7165) were assessed for eligibility through full-text review. In addition, the reference lists of these articles were screened, which led to a further 23 articles being identified. After full-text articles were assessed for eligibility, 89 reports were included in this scoping review for data charting and analysis (screening flow diagram in Figure 1). The basic information of the included articles is described as shown in Multimedia Appendix 6.

The included studies are predominantly observational, comprising 84 (94.38%) of the total. Among these, the longest cohort spanned 13 years [50]. One study explored relationships among up to 14,567 different diseases [51]. The largest sample size is derived from national-level integrated data infrastructure, which includes information from health bureaus and census networks [52]. Additionally, 13 (14.60%) studies focus on comorbidities, followed by 11 (12.36%) studies on diabetes and 10 (11.24%) studies each on cardiovascular diseases and cancers. The remaining studies cover an array of 20 different diseases.

### Word Frequency Analysis Summarizes the Types and Application Scenarios of Big Data–Driven Health Portraits

**Figure 2:**
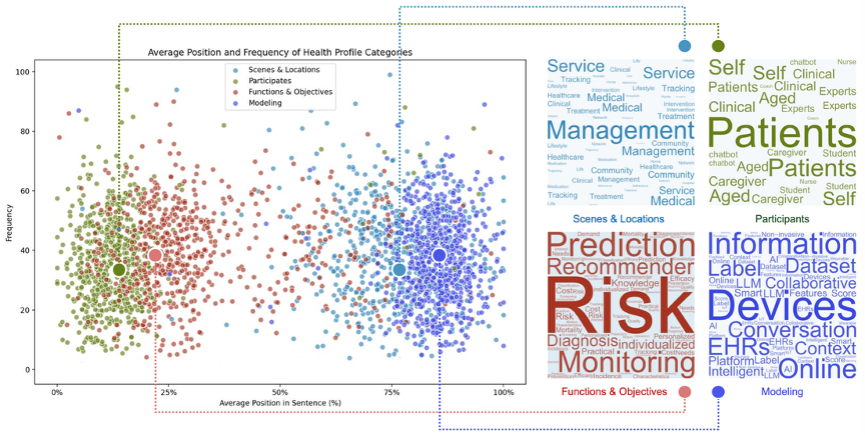
Word frequency analysis based on scatter plots. Extracted the keywords from the titles and abstracts.

The scatter plot (Figure 2) highlights key terms such as “management,” “medical,” “clinical,” “community,” “life,” “home,” and “trajectory,” reflecting the diverse application of health portraits in inpatient and outpatient settings for managing NCDs. Participants in these systems include medical professionals, patients, caregivers, and virtual health coaches, as indicated by terms like “patients,” “experts,” “caregiver,” “coach,” “chatbot,” “nurse,” and “self.” This underscores a collaborative approach spanning hospital care, community support, and patient self-management.

Health portraits support various NCD management functions, including risk management (“risk” and “prevention”), diagnosis and treatment (“diagnosis,” “incidence,” and “prediction”), disease progression and mortality stratification (“classification,” “prediction,” and “mortality”), health monitoring (“monitoring,” “tracking,” and “screening”), and providing tailored recommendations (“recommender,” “knowledge,” and “interest”).

High-frequency terms such as “individualized,” “personalized,” “efficacy,” and “cost” emphasize 2 primary goals: addressing unique patient needs and ensuring practical, cost-effective care. Additionally, terms like “information,” “device,” “EHRs,” and “dataset” indicate that health portrait modeling integrates data from diverse sources, including Internet of Things devices, wearables, digital platforms, EHRs, public datasets, and large language models (LLMs). This rich data foundation enables the extraction of relevant features and the creation of digital labels for comprehensive, individualized health portraits.

Through word frequency analysis and content summarization, this study identified 4 primary types of health portraits—diagnostic, prognostic, monitoring, and recommender—which collectively cover the health management continuum for NCDs. The different types of health portraits and their descriptions are shown in Table 2.

**Table 2:** Summary of different types of health portraits with management scenes based on full texts.

Type	Description	Management scene	Example
Diagnostic portrait	They are derived from clinical symptoms, signs, and laboratory test results, creating the labels related to patient diagnosis and treatment, and they can help doctors make accurate diagnoses in the early stages of the disease so that timely treatment measures can be taken [53].	Preclinical diagnosis and treatment prediction and preventive risk factor management based on the hospital environment and the medical team	Data were obtained from 9 independent datasets. Using predictors such as demographics, medical history, medication use, neuropsychological assessments, and multimodal neuroimaging, an artificial intelligence model based on a transformer architecture was developed for accurate etiological diagnosis and risk labeling in patients with dementia [54].
Prognostic portrait	They are derived from database information or relevant datasets, primarily consisting of a series of baseline characteristics of the patient, such as age, gender, stage of disease, and biomarkers, creating labels that reflect the health risks in the future [55].	Identification of high-risk patients with poor prognosis and management of risk factors for disease progression based on the in-hospital environment and the medical team	Data were obtained from the Big Data Center at Taipei Veterans General Hospital (VGH). Using predictors such as estimated glomerular filtration rate, hemoglobin, urine protein-to-creatinine ratio, insulin use, β-blocker use, renin-angiotensin system inhibitor use, and hypertension, a machine learning-based predictive model was developed to generate end-stage renal disease risk labels for patients with chronic kidney disease among sepsis survivors [56].
Monitoring portrait	They are derived from health care project processes and outcomes through self-reporting, mobile sensors, or wearables, with key labels derived from various performance metrics and descriptive summaries of patients [57,58].	Collaborative health management based on community and home scenarios, with the purpose of remote monitoring and management efficacy, with the participation of the health management team and patients	Data were obtained from obese adolescents participating in a weight management program. By analyzing interactions with Tess, an artificial intelligence–based behavioral coaching chatbot, a model was developed using natural language processing and machine learning to monitor and support weight management and prediabetes symptoms through digital health labeling [59].
Recommender portrait	They are derived from interactive information on digital platforms, focusing on using intelligent algorithms to recommend personalized information, resources, or interventions relevant to the user’s specific health needs [60].	Self-management based on community and home scenarios, centered on knowledge empowerment, and patient focus	Data were obtained from caregivers of patients with dementia recruited through social media advertisements. A knowledge graph–based dementia care intelligent recommendation system was developed using knowledge graph and intelligent recommendation system technologies to provide personalized care plan recommendations for patients with dementia and their caregivers [61].

### Data Usage of Big Data–Driven Health Portraits Based on the 3V Framework

**Figure 3:**
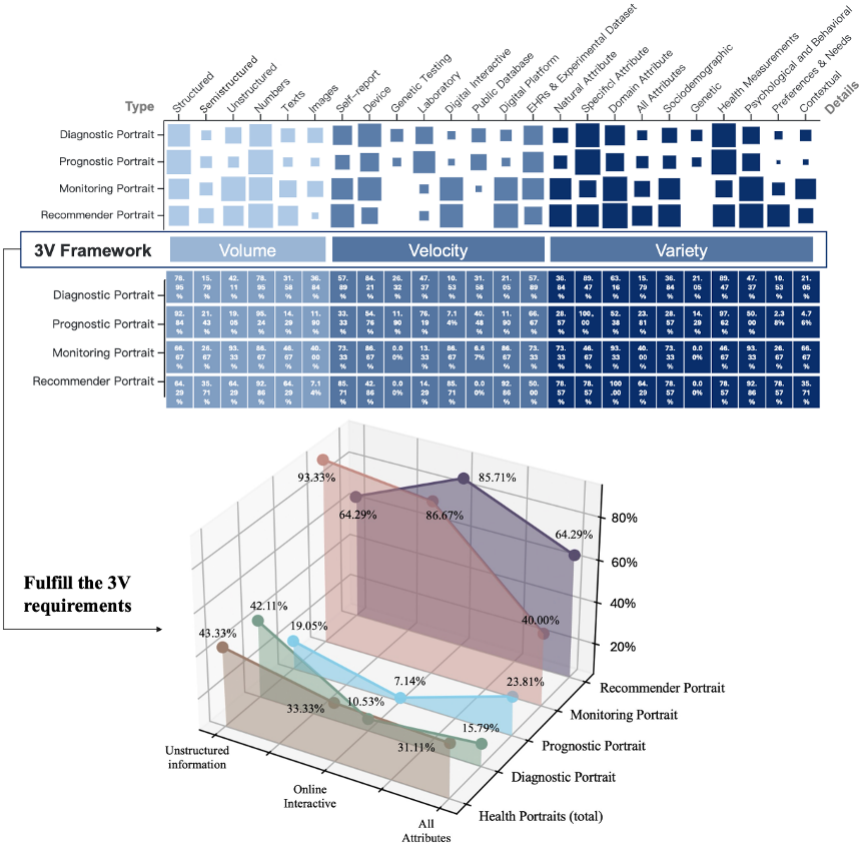
The heat map of data usage of health portraits based on the 3V (volume, velocity, and variety) framework. EHR: electronic health record.

Data usage is shown in Figure 3. Overall, only 17.78% of the included studies fulfill the requirements of big data based on the 3V framework. In terms of volume, structured data is highly used across the 4 types, with the lowest rate still reaching 64.29% in recommender portraits. However, the usage of unstructured data (43.33%) shows substantial variation: monitoring portraits lead with 93.33%, while prognostic portraits are at the low end with just 19.05%. Diagnostic portraits, focused similarly on clinical prediction models, exhibit a moderate usage of unstructured data at 42.11%. Regarding velocity, the reliance on digital interactive data (33.33%) is polarized. Diagnostic and prognostic portraits show lower usage rates, at 15.79% and 7.14%, respectively, whereas monitoring and recommender portraits both exceed 85%. For variety, only 31.11% of health portraits encompass all 3 attributes. However, recommender portraits demonstrate the highest performance, with 64.29% of studies encompassing all 3 attributes, among which domain attribute coverage is at 100%. Further, the domain attribute of recommender portraits primarily comes from preference-demand information, which constitutes 78.57% of its data—significantly higher than that in other portrait types. In contrast, prognostic portraits show the most limitations in the domain attribute. Although they excel in specialized medical data, reaching 100% in specific attribute coverage, they exhibit substantial gaps in preference-demand and contextual information, with usage rates of only 2.38% and 4.76%, respectively.

### Comprehensive Capability Assessment of Big Data–Driven Health Portraits Based on the 3V Framework and External Validation

**Figure 4:**
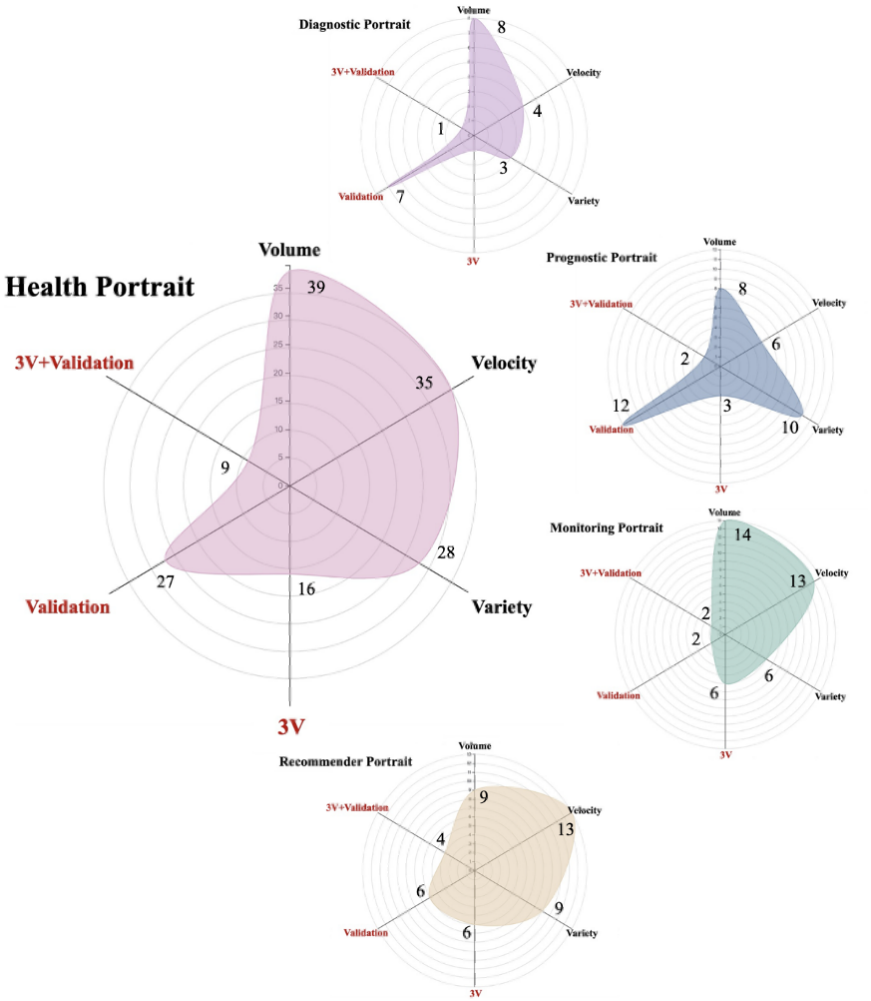
Comprehensive capability assessment radar charts that meet the needs of volume, velocity, and variety (3V)–based big data and external validation.

A comprehensive capability assessment was conducted in Figure 4. Overall, the external validation of the big data–driven health portraits is suboptimal, with only 27 studies (30.34%) meeting the required standards. Assessed together with each study's performance in meeting the 3V criteria of big data, only 9 (10.11%) studies meet these standards. Among the 4 types, recommender portraits showed relatively better results, with 4 (28.57%) studies meeting the comprehensive criteria. Finally, we conducted a comprehensive scoping review that synthesizes evidence from diverse literature on big data–driven health portraits, defining the comprehensive scope in Multimedia Appendix 7.

## Discussion

### Principal Results

Our findings indicate that big data–driven health portraits primarily cover the full spectrum of NCD management, categorized into 4 types, including diagnostic, prognostic, monitoring, and recommender portraits, each tailored to specific management scenarios. Despite the diversity of data sources for health portraits, a key limitation is the underuse of unstructured and interactive data, which restricts the potential of big data–driven approaches. A quantitative evaluation based on the “3V” framework further demonstrates weak comprehensive capabilities of big data–driven health portraits, largely due to insufficient use of diverse data types. Moreover, the lack of external validation metrics hampers the assessment of the generalizability of health portraits, and there is considerable room for improvement in validation efforts. This review emphasizes the need for continued exploration to enhance the adaptability and effectiveness of big data–driven health portraits in NCD management. The following sections address the key challenges, opportunities, and future roadmap, paving the way for innovative approaches tailored to specific management contexts.

The results showed that 4 primary types of health portraits span the care continuum from hospital-based to home-based settings. Specifically, diagnostic and prognostic portraits are predominantly deployed in clinical settings, where they leverage structured medical metrics and experimental data aligned with traditional health care models [62]. In contrast, monitoring and recommender portraits are increasingly applied in community and home-based contexts, supporting patient self-management, familial support, and interactions with health coaches [63,64]. Each type faces distinct data challenges. Hospital-based portraits benefit from high-quality data but lack continuity, while home-based systems provide continuous input via wearables and mobile devices, often with variable data quality [65]. Monitoring and recommender portraits, in particular, require diverse, high-quality training datasets and rigorous validation to ensure label accuracy and system reliability. Importantly, these types are not mutually exclusive—monitoring and recommender portraits offer real-time responsiveness, while diagnostic and prognostic portraits enhance predictive accuracy. Their integration presents opportunities for more comprehensive, adaptive health management. Emerging technologies, especially the integration of LLMs and knowledge graphs (KGs), are advancing this vision [14,66-70]. LLMs possess robust capabilities for processing unstructured and interactive data, such as chatbot dialogues [71], to extract real-time behavioral embeddings, while KGs structure multimodal biomarkers and social determinants into semantic networks and enable knowledge reasoning based on multimodal information. It must be recommended that this synergy will improve temporal resolution and contextual precision. Such architectures transcend traditional single-dimension health portraits, enabling adaptive health management across biological, behavioral, and environmental tiers [66,67,71]. Numerous experimental studies have shown that integrating LLMs with user historical interaction and behavioral information extracted from KGs can effectively perform relevant predictions and information recommendations. This method has exhibited superior effectiveness and reliability compared to traditional machine learning models across multiple public datasets [72-74]. However, significant challenges persist. Privacy and security concerns are particularly pressing, especially regarding the potential for data breaches during personal data interactions [71]. Addressing these concerns necessitates multifaceted approaches, including blockchain technology, end-to-end encryption, and advanced privacy protocols [75].

This study also reveals that while big data–driven health portraits demonstrate moderate performance across individual dimensions of the 3V framework, their overall effectiveness diminishes sharply when evaluated across all 3 dimensions simultaneously (16 (17.98%) studies). This suggests that health portraits are still in the early stages of development, where the comprehensiveness of data usage remains underdeveloped, which may constrain big data–driven health portraits in patent design or practical application. Upon closer inspection, there are considerable disparities in data use across the 4 types of health portraits. Diagnostic and prognostic portraits show significant differences in the use of unstructured data (volume). For instance, diagnostic portraits depend heavily on unstructured data like high-dimensional imaging, which encapsulates valuable predictive information [76-78], aligning with the findings of Esteva et al [79]. However, the prognostic model mostly used data from public databases, which provided structured self-reported data, though research has reported that integrating unstructured clinical text with structured data can improve model performance [80]. The possible reason for the problem is that, unlike the relatively clear diagnostic conditions and the relatively low requirements for data continuity, the prediction of prognostic outcomes depends on a large number of cohort data before and after treatment, and it is relatively difficult to obtain data from a single study in the real world, especially under the limited patient adherence [81-84]. Our findings also show that monitoring and recommender portraits excel in data volume, transmission, and variety, which may underscore the potential of wearable and contactless devices in health data capture [85-88] as a cost-effective way for all types of portraits to bridge the gaps in acquisition capability of unstructured and interactive data [80]. Moreover, from a portrait integration perspective, as previously discussed, the integration of KGs and LLMs offers a robust framework for achieving multimodal data fusion and health portrait unification [66,67,71]. The maturation of hardware devices supports cross-modal data interoperability, while the development of time-series fusion algorithms transforms conventional 2D labeling systems into dynamic 3D architectures [89,90]. By incorporating temporal granularity, our approach refines population-level subgroup features into individualized trajectory-aware markers, capturing health progression patterns from clinical to community settings. This evolution facilitates the organic unification of the 4 portrait types, thereby enabling precision health management plans tailored to individual developmental trajectories.

Our findings indicate that only 10.11% (9 studies) of studies meeting the “3V” criteria have undergone the external validation. This highlights a significant gap between theoretical potential and practical implementation, underscoring the need for more external validations in the real world. Unlike Youssef et al [91], who cautioned against the reliability of single models validated on limited datasets, our study emphasizes the importance of integrating external validation within a comprehensive big data framework. External validation is essential for assessing model generalizability and performance, supported by prior research [92-94]. We also found that diagnostic and prognostic models emphasize externally validated results [94]. One possible reason for this is the strong association between external validation and study quality evaluation recommended by *The BMJ’s* guidelines according to the “Prediction Model Risk of Bias Assessment Tool” (PROBAST) [95]. Another possible reason is that the reliability of the prediction results in the real world is the core of the value of model research, aiming to promote results-oriented resource allocation according to accurate risk levels to reduce costs and increase efficiency. Otherwise, models that lack external validation are at high risk of failure in real-world practice. Even though genotype metrics are considered a key component of precision medicine, with advantages such as high standardization, stability, and potential for causal inference, large-scale retrospective studies of predictive models based on genotype metrics have often failed [96]. This may be attributed to population heterogeneity and gene-environment interactions, which can diminish model performance in diverse populations in the real world [97]. Additionally, evaluations of 31 predictive models related to COVID-19 revealed that most studies had a high risk of bias [98]. Consequently, few are used or disseminated in clinical practice, thus failing to promote practical applications [99]. Monitoring and recommender portraits performed relatively poorly in external validation, probably because the guidance of these 2 types of models for health management was more process-oriented, such as patient intervention adherence or facilitating patients' resource usage in limited resources, so they focused more on data transmission speed and update frequency for the accuracy of external validation results in real-time monitoring [65,100]. Recent research has highlighted that intelligent predictive models extending beyond radiology and bioinformatics possess unique characteristics, which may need new evaluation tools such as PROBAST-AI, TRIPOD-AI (Transparent Reporting of a Multivariable Prediction Model for Individual Prognosis or Diagnosis–Artificial Intelligence) guidelines, and the criteria for health care conversation powered by AI [101,102]. Thus, future studies are recommended to use appropriate standards to complete external validation in real-world data to clarify the reliability of monitoring results and recommendations.

### Strengths and Limitations

To the best of our knowledge, this study is the first to conduct a scoping review of health portraits from a big data perspective, addressing a critical research gap and laying a solid theoretical foundation for future investigations. One of the key contributions is the introduction of the 3V framework, which operationalizes big data principles to quantify data usage, offering a novel perspective for evaluating health portrait development. Another contribution is that the structured analysis delineates the current state of the field and provides actionable strategies for theoretical advancements and practical applications. Furthermore, compared to traditional health portraits, this scope of big data–driven health portraits provides us with an opportunity to leverage multimodal data while grasping personal dynamic health status adaptively. Despite these contributions, several limitations must be acknowledged. First, restricting the review to English and Chinese studies may introduce language bias, potentially excluding valuable insights from other languages. Second, using a single binary classification metric for external validation limits the evaluation scope, making it difficult to pinpoint deficiencies in studies that do not meet benchmarks. Third, due to limitations in time and resources, this study lacks a more detailed analysis of health portrait features incorporating disease classifications, which will aid in developing a more comprehensive health portrait labeling system.

### Future Work

**Figure 5:**
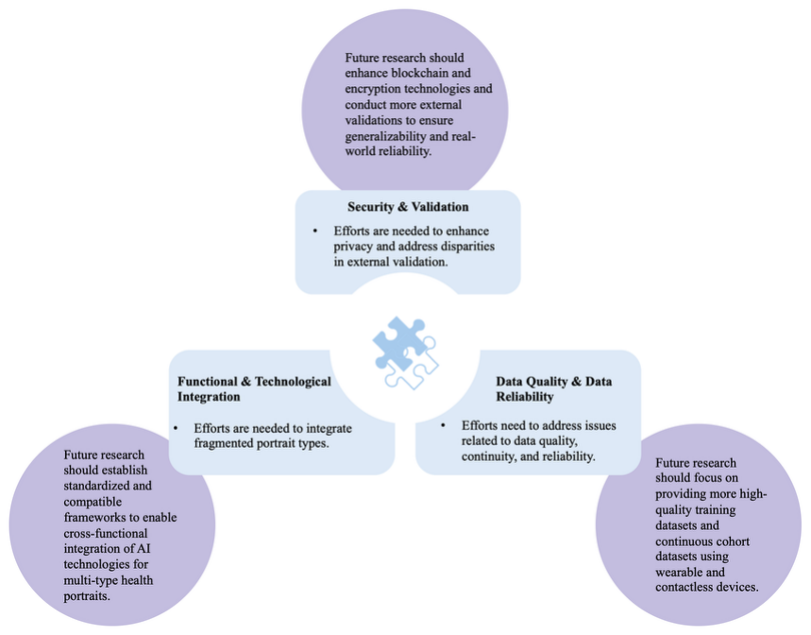
Challenges and future directions in big data–driven health portraits. A summary of the current challenges and possible future directions of big data–driven health portraits based on discussion and analysis of research results. AI: artificial intelligence.

This study underscores the challenges of big data–driven health portraits in NCD management in Figure 5, highlighting the possible future solutions that can fully realize the transformative potential. Among them, 2 main steps, according to our results, need to be highlighted: (1) prioritize LLM-driven integration of functionalities, contextual applications, and multisource data, while embedding ethical, privacy, and cost considerations at the design stage; and (2) promote more robust external validations according to appropriate standards. Notably, hyperpersonalization, which integrates multiomics data for tailored therapies, is highly recommended as a part of integrated health portraits [103-105]. With its convincing power in uncovering disease mechanisms, it brings the potential to integrate precision medicine with lifestyle nursing based on big data. Additionally, from a health equity perspective, future research could apply the PROGRESS-Plus (place of residence, race/ethnicity/culture/language, occupation, gender/sex, religion, education, socioeconomic status, and social capital) framework to health portraits, generating equity-based labels from natural attribute indicators to promote more equitable resource allocation and care for vulnerable populations [106]. Finally, building on our findings and previous research [18], we propose an integrated detailed roadmap for future practice as shown in Multimedia Appendix 8.

### Conclusions

Big data–driven health portraits offer significant potential to enhance personalized and precise management of NCDs. With the hardware advancements and cross-domain collaborations for holistic portrait design, chances are that the transformation of conventional 2D labeling systems into dynamic 3D architectures of integrated big data–driven health portraits through AI-driven approaches will reveal new opportunities. However, future research should focus on the privacy-utility tradeoffs and ethical dilemmas. Addressing these gaps will advance the development of personalized health management solutions.

## Appendix

Multimedia Appendix 1 PRISMA-ScR checklist.

Multimedia Appendix 2 Database-specific search strategy.

Multimedia Appendix 3 The principle and explanation of “Population,” “Concept,” and “Context.”.

Multimedia Appendix 4 Criteria for external validation.

Multimedia Appendix 5 The 3V (volume, velocity, and variety) framework and the comprehensive capability assessment.

Multimedia Appendix 6 Basic information about the included articles. An overview of articles included in the scoping review (N=90).

Multimedia Appendix 7 The scope of big data–driven health portraits in the management of noncommunicable diseases.

Multimedia Appendix 8 The roadmap of big data–driven health portraits in the management of noncommunicable diseases.

## Abbreviations

3V: volume, velocity, and variety
AI: artificial intelligence
EHR: electronic health record
KG: knowledge graph
LLM: large language model
NCD: noncommunicable disease
PRISMA-ScR: Preferred Reporting Items for Systematic Reviews and Meta-Analyses extension for Scoping Reviews
PROBAST: Prediction Model Risk of Bias Assessment Tool3V: volume, velocity, and variety
PROGRESS-Plus: place of residence, race/ethnicity/culture/language, occupation, gender/sex, religion, education, socioeconomic status, and social capital
TRIPOD-AI: Transparent Reporting of a Multivariable Prediction Model for Individual Prognosis or Diagnosis–Artificial Intelligence
