# Supplementary material for: Big Data–Driven Health Portraits for Personalized Management in Noncommunicable Diseases: Scoping Review
Source: J Med Internet Res. 2025 Jun 5;27:e72636. doi: 10.2196/72636 (PMC12179573; doi:10.2196/72636)
Supplement: Multimedia Appendix 2 [file jmir_v27i1e72636_app2.docx]

Table S2: Database-specific search strategy.

| Database | Search String | Last Search Date |
| --- | --- | --- |
| PubMed | (("Noncommunicable Diseases"[MeSH Terms] OR ("chronic diseases"[Title/Abstract] OR "noninfectious chronic diseases"[Title/Abstract])) AND ("User-Centered Design"[MeSH Terms] OR (("user"[All Fields] AND "profile*"[All Fields]) OR ("user"[All Fields] AND "persona*"[All Fields]) OR (("patient s"[All Fields] OR "patients"[MeSH Terms] OR "patients"[All Fields] OR "patient"[All Fields] OR "patients s"[All Fields]) AND "persona*"[All Fields]) OR (("patient s"[All Fields] OR "patients"[MeSH Terms] OR "patients"[All Fields] OR "patient"[All Fields] OR "patients s"[All Fields]) AND "profile*"[All Fields]) OR (("health"[MeSH Terms] OR "health"[All Fields] OR "health s"[All Fields] OR "healthful"[All Fields] OR "healthfulness"[All Fields] OR "healths"[All Fields]) AND ("portrait"[Publication Type] OR "portraits as topic"[MeSH Terms] OR "portrait"[All Fields])) OR ("user"[All Fields] AND "characteristic*"[All Fields]) OR "personaliz*"[All Fields] OR "tailor*"[All Fields] OR "individualiz*"[All Fields] OR ("individual s"[All Fields] OR "individualisation"[All Fields] OR "individualise"[All Fields] OR "individualised"[All Fields] OR "individualising"[All Fields] OR "individualism"[All Fields] OR "individualisms"[All Fields] OR "individualities"[All Fields] OR "individuality"[MeSH Terms] OR "individuality"[All Fields] OR "individualization"[All Fields] OR "individualize"[All Fields] OR "individualized"[All Fields] OR "individualizes"[All Fields] OR "individualizing"[All Fields] OR "individually"[All Fields] OR "individuals"[All Fields] OR "individuate"[All Fields] OR "individuated"[All Fields] OR "individuates"[All Fields] OR "individuating"[All Fields] OR "individuation"[MeSH Terms] OR "individuation"[All Fields] OR "individuations"[All Fields] OR "persons"[MeSH Terms] OR "persons"[All Fields] OR "individual"[All Fields]) OR ("precise"[All Fields] OR "precised"[All Fields] OR "precisely"[All Fields] OR "preciseness"[All Fields] OR "precises"[All Fields] OR "precision"[All Fields] OR "precisions"[All Fields]))) AND ("Big Data"[MeSH Terms] OR "Artificial Intelligence"[MeSH Terms] OR (("mega"[All Fields] AND ("data basel"[Journal] OR "brown univ dig addict theory appl"[Journal] OR "data"[All Fields])) OR ("large-scale"[All Fields] AND ("data basel"[Journal] OR "brown univ dig addict theory appl"[Journal] OR "data"[All Fields])) OR ("data science"[MeSH Terms] OR ("data"[All Fields] AND "science"[All Fields]) OR "data science"[All Fields]) OR ("high-dimensional"[All Fields] AND ("data basel"[Journal] OR "brown univ dig addict theory appl"[Journal] OR "data"[All Fields])) OR ("machine learning"[MeSH Terms] OR ("machine"[All Fields] AND "learning"[All Fields]) OR "machine learning"[All Fields])))) AND (2014:2025[pdat]) | 17-07-2024 |
| Embase | ('noncommunicable diseases':ab,ti OR 'chronic diseases':ab,ti OR 'noninfectious chronic diseases':ab,ti) AND ('user-centered design':ab,ti OR 'user profile':ab,ti OR 'user persona*':ab,ti OR 'patient profile*':ab,ti OR 'patient persona*':ab,ti OR 'health portrait*':ab,ti OR 'user characteristic*':ab,ti OR 'personaliz*':ab,ti OR 'tailor*':ab,ti OR 'individualiz*':ab,ti OR 'individuation':ab,ti OR 'precise':ab,ti) AND ('big data':ab,ti OR 'mega data':ab,ti OR 'large-scale data':ab,ti OR 'data science':ab,ti OR 'high-dimensional data':ab,ti OR 'machine learning':ab,ti OR 'artificial intelligence':ab,ti) | 17-07-2024 |
| Scopus | ( TITLE-ABS-KEY ( "big data" OR "large-scale data" OR "high-dimensional data" OR "data science" OR "machine learning" OR "artificial intelligence" OR "analytics" ) AND TITLE-ABS-KEY ( "noncommunicable diseases" OR "chronic diseases" OR "noninfectious chronic diseases" ) AND TITLE-ABS-KEY ( "user-centered design" OR "user profile" OR "user persona*" OR "health portrait*" OR "user characteristic*" OR "personaliz*" OR "tailor*" OR "individualiz*" OR "individuation" OR "precise" ) ) AND PUBYEAR > 2013 | 17-07-2024 |
| EBSCO | (TI "big data" OR TI "large-scale data" OR TI "data science" OR TI "high-dimensional data" OR TI "machine learning" OR TI "artificial intelligence" OR TI "analytics" OR AB "big data" OR AB "large-scale data" OR AB "data science" OR AB "machine learning" OR AB "artificial intelligence" OR AB "analytics")  AND  (TI "noncommunicable diseases" OR TI "chronic diseases" OR TI "noninfectious chronic diseases" OR AB "noncommunicable diseases" OR AB "chronic diseases" OR AB "noninfectious chronic diseases")  AND  (TI "user-centered design" OR TI "user profile" OR TI "user persona*" OR TI "health portrait*" OR TI "user characteristic*" OR TI "personaliz*" OR TI "tailor*" OR TI "individualiz*" OR TI "individuation" OR TI "precise" OR AB "user-centered design" OR AB "user profile" OR AB "user persona*" OR AB "health portrait*" OR AB "user characteristic*" OR AB "personaliz*" OR AB "tailor*" OR AB "individualiz*" OR AB "individuation" OR AB "precise")  AND DT 20140101-20240717 | 17-07-2024 |
| Ovid | 1 (noncommunicable diseases or Chronic diseases or Noninfectious chronic diseases).mp. [mp=title, abstract, full text, caption text]  2 (Personaliz* or Tailor* or individualiz* or individuation or precise User-centered Design or user profile* or user persona* or patient persona* or patient profile* or health portrait or user characteristic*).mp. [mp=title, abstract, full text, caption text]  3 (big data or mega data or large-scale data or data science or high-dimensional data or machine learning or artificial intelligence or analytics).mp. [mp=title, abstract, full text, caption text]  4 1 and 2 and 3  5 limit 4 to yr="2014 -Current" | 17-07-2024 |
| Springer Nature Link | ('noncommunicable diseases' OR 'chronic diseases' OR 'noninfectious chronic diseases') AND ('user-centered design' OR 'user profile' OR 'user persona*' OR 'patient profile*' OR 'patient persona*' OR 'health portrait*' OR 'user characteristic*' OR 'personaliz*' OR 'tailor*' OR 'individualiz*' OR 'individuation' OR 'precise') AND ('big data' OR 'mega data' OR 'large-scale data' OR 'data science' OR 'high-dimensional data' OR 'machine learning' OR 'artificial intelligence') | 17-07-2024 |
| Web of science | (((AB=(noncommunicable diseases OR Chronic disease OR Noninfectious chronic diseases)) AND AB=(Personaliz* OR Tailor* OR individualiz* OR individuation OR precise OR User-centered Design OR user profile* OR user persona* OR patient persona* OR patient profile* OR health portrait OR user characteristic*)) AND AB=(big data OR mega data OR large-scale data OR data science OR high-dimensional data OR machine learning OR artificial intelligence OR analytics)) | 17-07-2024 |
