# Supplementary material for: Big Data–Driven Health Portraits for Personalized Management in Noncommunicable Diseases: Scoping Review
Source: J Med Internet Res. 2025 Jun 5;27:e72636. doi: 10.2196/72636 (PMC12179573; doi:10.2196/72636)
Supplement: Multimedia Appendix 3 [file jmir_v27i1e72636_app3.docx]

Table S3: The principle and explanation of “Population,” “Concept,” and “Context”.

| Definition Adopted in This Study | | Additional Notes |
| --- | --- | --- |
| Population | | |
|  | The study population is limited to NCDs, primarily including cardiovascular diseases, cancer, diabetes, and chronic respiratory diseases as defined by the WHO[1]. | None |
|  | Considering the concept of health portraits centered on feature identification and patient stratification, we also included studies on a broader range of NCDs where relevant, like obesity, mental disorders, and geriatric syndromes to ensure a comprehensive review of health portrait types and the methodologies employed[2,3]. |  |
| Concept | | |
|  | The included articles had to report about health portraits, like the methodology of modelling for patient clustering or stratification. | Since we found that limiting the search term to 'health portrait' or ‘health profile’ yielded relatively few results, we expanded the search terms, according to relevant reviews and iterative literature research. |
|  | We included studies related to health portraits, user-centered design, predictive models, recommendation systems, mHealth, and decision support, as well as research on intelligent, personalized, and precision medicine. After the initial search, portraits were categorized by content induction. For the types with fewer studies, we continued the secondary search to achieve methodological saturation and summarization. |  |
| Context | | |
|  | Studies included were required to meet big data standards, typically involving large volumes and complex computational analysis. | While data science (DS) may require datasets in the terabytes or petabytes, a high speed of generating or processing data, and variety of data format like structured, semi-structured, and unstructured types (text, audio, and video), this study were not strictly applied. |
|  | The search strategy used terms like 'big data' to capture studies with dynamic, multi-dimensional datasets. | We broadened the scope of 'big data' in healthcare to exclude studies relying solely on traditional questionnaires or qualitative methods. |
|  | Although no strict thresholds for sample size or speed were set, we still focused on the 3Vs of big data—volume, variety, and velocity—to assess the dynamic potential of each methodology in an innovative way. Indeed, recommendations from experts helped refine our definitions: volume focused on data structure and forms; velocity emphasized data source diversity; and variety addressed content. | For the ‘Volume’, medical studies often measure data by patients or sample size, making direct comparisons challenging. |
|  |  | For the ‘Velocity’, the generation and transmission of health data—often dependent on professional medical devices, wearable devices or clinical reports—can be slow and discontinuous, relying on the patient's initiative. Besides, it remains security and ethical concerns in medical data transmission and usage right which poses unique challenges. |
|  |  | For the ‘Variety’, it must be highlighted that there was a mismatch between data volume and information density in NCDs care. For example, smoking behavior in medical data is not just a lifestyle habit but is intricately linked to health risks, such as acute conditions[4,5]. |

1. Noncommunicable diseases. Accessed October 9, 2024. https://www.who.int/news-room/fact-sheets/detail/noncommunicable-diseases

2. The path to healthy ageing in China: a Peking University–Lancet Commission. *The Lancet*. 2022;400(10367):1967-2006. doi:10.1016/S0140-6736(22)01546-X

3. J Z, C S, Z L, et al. Burden of noncommunicable diseases among children and adolescents aged 10-24 years in China, 1990-2019: A population-based study. *Cell reports Medicine*. 2023;4(12). doi:10.1016/j.xcrm.2023.101331

4. Jin Jiqiong, Ju Lei, Zhang Yi, Si Yong, Wang Wenjun. Character identification and value assessment of cigarette consumers based on consumer persona. *Tobacco Science & Technology*. 2023;56(1):105-112.

5. Ding N, Sang Y, Chen J, et al. Cigarette Smoking, Smoking Cessation, and Long-Term Risk of 3 Major Atherosclerotic Diseases. *Journal of the American College of Cardiology*. 2019;74(4):498-507. doi:10.1016/j.jacc.2019.05.049
