# Supplementary material for: Big Data–Driven Health Portraits for Personalized Management in Noncommunicable Diseases: Scoping Review
Source: J Med Internet Res. 2025 Jun 5;27:e72636. doi: 10.2196/72636 (PMC12179573; doi:10.2196/72636)
Supplement: Multimedia Appendix 4 [file jmir_v27i1e72636_app4.docx]

Table S4: The criteria for external validation

| Type | Criteria | Explanation |
| --- | --- | --- |
| **Clinical predictive models** | | |
|  | **Yes**: validation only or development and validation within the same publication | For predictive models in observational studies, they first determine if external validation has been performed and then assess its validity based on the study content. Following BMJ guidelines, specific criteria are set according to the “Prediction Model Risk of Bias Assessment Tool” (PROBAST)[1]. In PROBAST, models are categorized for each evaluation as "development only," "development and validation within the same publication," or "validation only." If a publication focuses on creating a model by adding one or more predictors to an established model or set of predictors, it should be classified as "development only." Publications that validate an existing model on separate data and subsequently update (refine or extend) it, effectively developing a new model, fall under "development and validation within the same publication." Note that a single publication may address multiple models of interest. Studies included in the review were classified accordingly: "validation only" or "development and validation within the same publication" were marked as "Yes" for external validation, while "development only" was marked as "No." |
|  | **No**: development only |  |
| **Health recommender systems** | | |
|  | **Yes**: evaluations involving end users | For health recommender system in observational studies, the researchers review the full text to determine if this study include evaluations involving end users, to ensure the safety, clinical effectiveness, patient perspectives, economic aspects, sociocultural, ethical, and legal aspects[2]. |
|  | **No**: evaluations used metrics to assess performance (no user) |  |
| **Interventional studies** | | |
|  | **Yes**: effective | For interventional studies, the researchers review the full text to clarify the intervention’s effectiveness and determine whether it was effective. |
|  | **Yes**: effective  **No**: not effective |  |

Reference

1. Wolff RF, Moons KGM, Riley RD, et al. PROBAST: A Tool to Assess the Risk of Bias and Applicability of Prediction Model Studies. *Ann Intern Med*. 2019;170(1):51-58. doi:10.7326/M18-1376

2. Sun Y, Zhou J, Ji M, Pei L, Wang Z. Development and Evaluation of Health Recommender Systems: Systematic Scoping Review and Evidence Mapping. *J Med Internet Res*. 2023;25:e38184. doi:10.2196/38184
