# Supplementary material for: Big Data–Driven Health Portraits for Personalized Management in Noncommunicable Diseases: Scoping Review
Source: J Med Internet Res. 2025 Jun 5;27:e72636. doi: 10.2196/72636 (PMC12179573; doi:10.2196/72636)
Supplement: Multimedia Appendix 5 [file jmir_v27i1e72636_app5.docx]

Table S5: The 3V framework

| Field-I | Field-II | Recommended | Details |
| --- | --- | --- | --- |
| Volume | | | |
|  | Unstructured information | Yes |  |
|  | Numbers | Yes |  |
|  | Characters | Yes |  |
|  | images | Yes |  |
| Velocity | | | |
|  | Self-report | Yes |  |
|  | Device | Yes |  |
|  | Genetic testing | Yes |  |
|  | Laboratory | Yes |  |
|  | Online interactive | Yes |  |
| Variety | | | |
|  | Natural attribute | Yes | Sociodemographic |
|  | Specific attribute | Yes | Genetic |
|  |  |  | Health Measurements |
|  | Domain attribute | Yes | Behavioral Needs |
|  |  |  | Preferences |
|  |  |  | Contextual |

Yes: When a portrait model fulfills all these criteria, it is deemed to a robust big data-driven health portrait: to meet the "unstructured data use" in Volume, the "digitalized interactive platform" in Velocity, and covers the three attributes of natural, domain-specific, and specific in Variety.

Supplementary Table S6: The comprehensive capability assessment

|  | Utility evaluation | 3V framework | | |
| --- | --- | --- | --- | --- |
|  |  | Volume | Velocity | Variety |
| Recommended | Yes | Yes | Yes | Yes |
| Record |  |  |  |  |

Yes: When a portrait model fulfills all these criteria, it is deemed to a robust big data-driven health portrait: to meet the "unstructured data use" in Volume, the "digitalized interactive platform" in Velocity, and covers the three attributes of natural, domain-specific, and specific in Variety. Utility evaluation assesses whether the portrait model has undergone external validation.
