# Supplementary material for: Big Data–Driven Health Portraits for Personalized Management in Noncommunicable Diseases: Scoping Review
Source: J Med Internet Res. 2025 Jun 5;27:e72636. doi: 10.2196/72636 (PMC12179573; doi:10.2196/72636)
Supplement: Multimedia Appendix 6 [file jmir_v27i1e72636_app6.docx]

Table S7: The basic information of the included articles. The overview of articles included in the scoping review (N=90)

| Author | Year | Country | Design | Participant | Not ML | ML | IA | AI-driven | Others | External Validation | Sample Size |
| --- | --- | --- | --- | --- | --- | --- | --- | --- | --- | --- | --- |
| Duan et al[1] | 2024 | China | observational study | elderly health issues |  |  |  | Yes |  | No | 465 |
| Hu et al[2] | 2021 | China | observational study | DM |  |  | Yes |  |  | No | 1097 |
| Wang et al[3] | 2018 | China | observational study | elderly health issues |  |  |  | Yes |  | No | NA |
| Kang et al[4] | 2019 | China | observational study | HTN |  |  | Yes |  |  | No | 61123 |
| Kwon et al[5] | 2019 | Korea | observational study | HF |  | Yes |  |  |  | Yes | 6924 |
| Kuo et al[6] | 2019 | China | observational study | CKD |  |  | Yes |  |  | No | 1297 |
| Tsai et al[7] | 2019 | China | observational study | CKD | Yes |  |  |  |  | No | 4647 |
| Ye et al[8] | 2018 | USA | observational study | HTN |  | Yes |  |  |  | No | 823627 |
| Lin et al[9] | 2019 | China | observational study | COPD |  | Yes |  |  |  | No | 186188 |
| Srinivas et al[10] | 2017 | USA | observational study | post-transplant kidney disease issues | Yes |  |  |  |  | No | 1349 |
| Maxwell et al[11] | 2017 | USA | observational study | chronic disease | Yes |  |  |  |  | No | 110300 |
| Razavian et al[12] | 2015 | USA | observational study | DM |  | Yes |  |  |  | No | 4100000 |
| Schüssler-Fiorenza et al[13] | 2019 | USA | observational study | prediabetes |  |  | Yes |  |  | No | 109 |
| Oh et al[14] | 2016 | USA | observational study | DM |  | Yes |  |  |  | No | 43509 |
| Lei et al[15] | 2023 | China | observational study | lung cancer | Yes |  |  |  |  | No | 3398 |
| Lv et al[16] | 2022 | China | observational study | mental disorder | Yes |  |  |  |  | No | 773 |
| Cai et[17] | 2022 | China | observational study | GBC, CC |  | Yes |  |  |  | No | 15 |
| Mu et al[18] | 2021 | China | observational study | DM, HTN |  | Yes |  |  |  | Yes | 14579 |
| Yang et[19] | 2021 | China | observational study | HTN, CHD |  | Yes |  |  |  | No | 71120 |
| Liu et al[20] | 2022 | China | observational study | DM |  | Yes |  |  |  | No | 1258 |
| Cao et al[21] | 2023 | China | observational study | DM |  | Yes |  |  |  | Yes | 2424 |
| Liu et al[22] | 2023 | China | observational study | NCD |  | Yes |  |  |  | No | 32448 |
| Zhang et al[23] | 2023 | USA | observational study | AD |  |  | Yes |  |  | No | NA |
| Wang et al[24] | 2023 | China | observational study | chronic disease |  |  | Yes |  |  | Yes | 14063 |
| Liu et al[25] | 2021 | China | observational study | elderly health issues |  |  | Yes |  |  | No | 222 |
| Lee et al[26] | 2022 | China | observational study | CKD |  | Yes |  |  |  | No | 11661 |
| Zhang et al[27] | 2021 | China | observational study | chronic disease |  |  | Yes |  |  | Yes | 43156 |
| Han et al[28] | 2023 | China | observational study | ESCC |  | Yes |  |  |  | Yes | 104129 |
| Jiang et al[29] | 2022 | China | observational study | DM |  | Yes |  |  |  | No | NA |
| Hu et al[30] | 2022 | China | observational study | chronic disease |  | Yes |  |  |  | No | 1100000 |
| Fujihara et al[31] | 2023 | Japan | observational study | obesity |  | Yes |  |  |  | No | 55000 |
| Kweon et al[32] | 2020 | USA | observational study | cancer |  | Yes |  |  |  | No | 9538 |
| Chen et al[33] | 2024 | UK | observational study | chronic pain issues | Yes |  |  |  |  | No | 24405 |
| Ren et al[34] | 2022 | China | observational study | DKD |  | Yes |  |  |  | No | 890 |
| Lee et al[35] | 2023 | USA | observational study | CKD | Yes |  |  |  |  | No | 11495668 |
| King et al[36] | 2021 | China | observational study | CKD | Yes |  |  |  |  | No | 7135 |
| Yu et al[37] | 2022 | China | observational study | breast cancer |  |  | Yes |  |  | Yes | 603 |
| Zhang et al[38] | 2022 | USA | observational study | chronic disease |  |  |  |  | Yes | Yes | 4278 |
| Chandran et al[39] | 2023 | USA | observational study | lung cancer |  | Yes |  |  |  | Yes | 4777606 |
| Song et al[40] | 2020 | USA | observational study | T2DM, CKD |  | Yes |  |  |  | No | 14039 |
| Tavakoli et al[41] | 2020 | Canada | observational study | COPD |  | Yes |  |  |  | No | 222219 |
| Surian et al[42] | 2024 | Singapore | observational study | T2DM |  |  |  |  | Yes | Yes | 7072 |
| Chen et al[43] | 2022 | USA | observational study | CHF, CKD | Yes |  |  |  |  | No | 4638 |
| Shen et al[44] | 2024 | USA | observational study | breast cancer, ovarian cancer |  | Yes |  |  |  | Yes | 2895 |
| Chen et[45] | 2023 | USA | observational study | AECOPD |  | Yes |  |  |  | Yes | 3660 |
| Alikhan et al[46] | 2023 | India | observational study | CKD |  | Yes |  |  |  | No | 400 |
| Kluckert et al[47] | 2024 | Switzerland | observational study | suspected prostate cancer |  |  | Yes |  |  | Yes | 832 |
| Vetrano et al[48] | 2022 | USA | observational study | UC, atopic dermatitis |  | Yes |  |  |  | Yes | 500 |
| Kuhlemeier et al[49] | 2022 | USA | interventional study | obesity |  | Yes |  |  |  | Yes | 991 |
| Pretzsch et al[50] | 2022 | Australia | observational study | autism | Yes |  |  |  |  | No | 2073 |
| Burger et al[51] | 2023 | Sweden | observational study | HF | Yes |  |  |  |  | Yes | 66701 |
| Hageman et al[52] | 2023 | Netherlands | observational study | ASCVD |  | Yes |  |  |  | No | 8355 |
| Maher et al[53] | 2020 | Australia | interventional study | obesity, overweight |  |  |  | Yes |  | Yes | 99 |
| Shed et al[54] | 2019 | USA | observational study | breast cancer |  |  |  | Yes |  | Yes | 958 |
| Stephens et al[55] | 2019 | the USA | interventional study | obesity, pre-DM |  |  |  | Yes |  | Yes | 23 |
| Stein et al[56] | 2017 | the USA | interventional study | obesity, overweight |  |  |  | Yes |  | Yes | 70 |
| Barbieri et al[57] | 2022 | Australia | observational study | high risk of CVD |  | Yes |  |  |  | No | 2164872 |
| Xue et al[58] | 2024 | the USA | observational study | cerebrovascular disease |  | Yes |  |  |  | Yes | 51269 |
| Fang et al[59] | 2016 | the USA | observational study | NCD |  | Yes |  |  |  | No | 20927 |
| Kosvyra et al[60] | 2020 | Greece | observational study | CLL |  | Yes |  |  |  | Yes | 26 |
| Patro et al[61] | 2021 | the USA | observational study | CVD |  | Yes |  |  |  | No | 294 |
| Kumar et al[62] | 2024 | India | observational study | ADHF |  | Yes |  |  |  | No | 151 |
| Liu et al[63] | 2020 | China | observational study | COPD, RHD |  | Yes |  |  |  | Yes | 1261 |
| Chung et al[64] | 2019 | Korea | observational study | NCD |  |  | Yes |  |  | No | NA |
| Hossain et al[65] | 2023 | Several countries in North America | observational study | CVD, high risk of CVD |  | Yes |  |  |  | No | 918 |
| Feng et al[66] | 2023 | the USA | observational study | AKI |  | Yes |  |  |  | No | 4375 |
| Trastulla et al[67] | 2024 | the UK | observational study | CAD, SCZ |  | Yes |  |  |  | Yes | 367620 |
| Kim et al[68] | 2015 | Korea | observational study | chronic disease |  |  |  | Yes |  | NA | NA |
| Hu et al[69] | 2021 | the USA | observational study | high risk of lung cancer |  | Yes |  |  |  | No | 50062 |
| Chaix et al[70] | 2019 | France | observational study | breast cancer |  |  |  | Yes |  | Yes | 4737 |
| Montagna et al[71] | 2024 | Italy | observational study | HTN |  |  |  | Yes |  | No | NA |
| Kim et al[68] | 2020 | Korea | observational study | chronic disease |  |  | Yes |  |  | No | 197 |
| Agrawal et al[72] | 2020 | the USA | observational study | DM |  | Yes |  |  |  | No | 867 |
| Nghiem et al[73] | 2023 | New Zealand | observational study | CVD |  | Yes |  |  |  | No | NA |
| Li et al[74] | 2022 | Several countries in Europe | observational study | Ischemic stroke |  | Yes |  |  |  | No | 1756 |
| Ye et al[75] | 2020 | the USA | observational study | DM |  | Yes |  |  |  | No | 9954 |
| Murugesh et al[76] | 2024 | Several countries in Europe | observational study | AD, MCI |  | Yes |  |  |  | No | 1311 |
| Safa et al[77] | 2023 | India | observational study | CVD |  |  | Yes |  |  | No | NA |
| Mogaveera et al[78] | 2021 | India | observational study | DM, HTN, thyroid disease |  | Yes |  |  |  | No | NA |
| Jin et al[79] | 2024 | the USA | interventional study | metabolic health risks |  |  | Yes |  |  | Yes | 16 |
| Silişteanu et al[80] | 2015 | Romania | interventional study | chronic low back pain |  | Yes |  |  |  | Yes | 175 |
| Gupta et al[81] | 2022 | the USA | observational study | mental health issues |  |  | Yes |  |  | NA | NA |
| Kathan et al[82] | 2022 | Germany | observational study | depression |  |  | Yes |  |  | No | 65 |
| Adams et al[83] | 2014 | the USA | observational study | stress | Yes |  |  |  |  | NA | 7 |
| Preran et al[84] | 2021 | the USA | observational study | depression |  | Yes |  |  |  | No | 138 |
| Fukazawa et al[85] | 2019 | Japan | observational study | stress |  | Yes |  |  |  | No | 20 |
| Kanjo et al[86] | 2017 | the UK | observational study | affective states |  | Yes |  |  |  | No | 50 |
| Jacobson et al[87] | 2020 | the USA | observational study | depression |  | Yes |  |  |  | No | 31 |
| O'Brien et al[88] | 2017 | the UK | observational study | depression | Yes |  |  |  |  | NA | 59 |
| Matteo et al[89] | 2021 | Canada | observational study | depression, stress |  | Yes |  |  |  | No | 84 |

**Abbreviations**

HTN: Hypertension

DM: Diabetes

CHD: Coronary Heart Disease

CVD: Cardiovascular Diseases

ASCVD: Atherosclerotic Cardiovascular Disease

COPD: Chronic Obstructive Pulmonary Disease

AECOPD: Acute Exacerbation of Chronic Obstructive Pulmonary Disease

ESCC: Esophageal Squamous Cell Carcinoma

RHD: rheumatic heart disease

SCZ: Schizophrenia

MCI: Mild Cognitive Impairment

CKD: Chronic Kidney Disease

ADHF: Acute Decompensated Heart Failure

CHF: Chronic Heart Failure

HF: Heart Failure

CLL: Early Chronic Lymphocytic Leukemia

AD: Alzheimer's disease

NCD: Non-communicable Disease

GBC: Gallbladder Cancer

CC: Cholangiocarcinoma

UC: Ulcerative Colitis

T2DM: Type 2 Diabetes

DKD: Diabetic Kidney Disease

ML: Machine Learning

IA: Intelligence Augmented

AI: Artificial Intelligence

**References**

1. Duan J. *Research on Accurate Recommendation Service for Elderly Users in Online Health Community Based on User Portraits*. Master. Heilongjiang University; 2024. https://kns.cnki.net/kcms2/article/abstract?v=axnrJTP8flxnRgBBK89PDiRhCkLuDcsxshsVOSO0kw77bxz5fCr0vk6zmV3xECmD6kGmkM7uP4AZos_f93SHqmu4WBfF9x57V9AwvVD3WewPw0TKnkQPEQ==&uniplatform=NZKPT&language=gb

2. Hu P. *Research on Scenario-based Information Recommendation of Chronic Disease Online Health Communities Based on User Portraits*. Master. Central China Normal University; 2021. https://kns.cnki.net/kcms2/article/abstract?v=smPsKIJgVaBMcl-lc0rfCm0PyLq_uX6yOSbLYJKrS1OXwEr7URf9nrqMLSW24NSfAh19U_hNJc14Eov1-x2nvSNvXhmEFKvdOGZm34WUuA1y0sSB1yxtYw==&uniplatform=NZKPT&language=gb

3. Wang X. Research on Personalized Knowledge Recommendation Service Modeling Based on Agent. *Dongbei University of Finance and Economics*. 2017;(07).

4. Kang F. Individualized efficacy evaluation of hypertensive drugs in the real-world community population. *Shandong University*. 2019;(09).

5. Kwon JM, Kim KH, Jeon KH, et al. Artificial intelligence algorithm for predicting mortality of patients with acute heart failure. *PLoS One*. 2019;14(7):e0219302. doi:10.1371/journal.pone.0219302

6. Kuo CC, Chang CM, Liu KT, et al. Automation of the kidney function prediction and classification through ultrasound-based kidney imaging using deep learning. *NPJ Digit Med*. 2019;2:29. doi:10.1038/s41746-019-0104-2

7. Tsai CW, Huang HC, Chiang HY, et al. Longitudinal lipid trends and adverse outcomes in patients with CKD: a 13-year observational cohort study. *J Lipid Res*. 2019;60(3):648-660. doi:10.1194/jlr.P084590

8. Ye C, Fu T, Hao S, et al. Prediction of Incident Hypertension Within the Next Year: Prospective Study Using Statewide Electronic Health Records and Machine Learning. *J Med Internet Res*. 2018;20(1):e22. doi:10.2196/jmir.9268

9. Lin S, Zhang Q, Chen F, Luo L, Chen L, Zhang W. Smooth Bayesian network model for the prediction of future high-cost patients with COPD. *Int J Med Inform*. 2019;126:147-155. doi:10.1016/j.ijmedinf.2019.03.017

10. Srinivas TR, Taber DJ, Su Z, et al. Big Data, Predictive Analytics, and Quality Improvement in Kidney Transplantation: A Proof of Concept. *Am J Transplant*. 2017;17(3):671-681. doi:10.1111/ajt.14099

11. Maxwell A, Li R, Yang B, et al. Deep learning architectures for multi-label classification of intelligent health risk prediction. *BMC Bioinformatics*. 2017;18(Suppl 14):523. doi:10.1186/s12859-017-1898-z

12. Razavian N, Blecker S, Schmidt AM, Smith-McLallen A, Nigam S, Sontag D. Population-Level Prediction of Type 2 Diabetes From Claims Data and Analysis of Risk Factors. *Big Data*. 2015;3(4):277-287. doi:10.1089/big.2015.0020

13. Schüssler-Fiorenza Rose SM, Contrepois K, Moneghetti KJ, et al. A longitudinal big data approach for precision health. *Nat Med*. 2019;25(5):792-804. doi:10.1038/s41591-019-0414-6

14. Oh W, Kim E, Castro MR, et al. Type 2 Diabetes Mellitus Trajectories and Associated Risks. *Big Data*. 2016;4(1):25-30. doi:10.1089/big.2015.0029

15. Lei H, Tao D, Zhang N, et al. Nomogram prediction for the risk of venous thromboembolism in patients with lung cancer. *Cancer Cell Int*. 2023;23(1):40. doi:10.1186/s12935-023-02882-1

16. Lv Y, Zhao H, Wu J. Behavioral profile of patients with mental disorders in online health community. *Chinese Mental Health Journal*. 2022;36(5):385-390.

17. Cai C. User Portrait Research on Online Health Community Based on User Behavior Indices. *Modern Information Technology*. 2022;6(6):144-147. doi:10.19850/j.cnki.2096-4706.2022.06.037

18. Mu D. Chronic disease prediction and visual analysis based on medical health data. *Southwest University*. 2021;(08). doi:10.27415/d.cnki.gxngc.2021.000967

19. Yang Y. Accurate Modeling of Cardiovascular Chronic Disease Risk Based on Medical Big Data. 2021;(01). doi:10.27822/d.cnki.gszxj.2021.000012

20. Liu Q. RESEARCH AND IMPLEMENTATION OF PERSONALIZED DIET RECOMMENDATION FOR CHRONIC DISEASE MANAGEMENT. *Harbin Institute of Technology*. Published online 2022. doi:10.27061/d.cnki.ghgdu.2022.002748

21. Cao J, Zhong Y, Zou N, Yao Y, Cai L. Research on Personalized Temporal Feature Fusion Recommendation Algorithm fot Online Health Community. *Journal of Modern Information*. 2023;43(9):26-35.

22. Liu M, Zhou J, Xi Q, et al. A computational framework of routine test data for the cost-effective chronic disease prediction. *Brief Bioinform*. 2023;24(2). doi:10.1093/bib/bbad054

23. Zhang X, Li Z, Zhang Q, Yin Z, Lu Z, Li Y. A new weakly supervised deep neural network for recognizing Alzheimer’s disease. *Comput Biol Med*. 2023;163:107079. doi:10.1016/j.compbiomed.2023.107079

24. Wang J, Gao Y, Wang F, et al. Accurate estimation of biological age and its application in disease prediction using a multimodal image Transformer system. *Proc Natl Acad Sci U S A*. 2024;121(3):e2308812120. doi:10.1073/pnas.2308812120

25. Liu Y, He X, Wang R, et al. Application of Machine Vision in Classifying Gait Frailty Among Older Adults. *Front Aging Neurosci*. 2021;13:757823. doi:10.3389/fnagi.2021.757823

26. Lee KH, Chu YC, Tsai MT, et al. Artificial Intelligence for Risk Prediction of End-Stage Renal Disease in Sepsis Survivors with Chronic Kidney Disease. *Biomedicines*. 2022;10(3):546. doi:10.3390/biomedicines10030546

27. Zhang K, Liu X, Xu J, et al. Deep-learning models for the detection and incidence prediction of chronic kidney disease and type 2 diabetes from retinal fundus images. *Nat Biomed Eng*. 2021;5(6):533-545. doi:10.1038/s41551-021-00745-6

28. Han J, Guo X, Zhao L, et al. Development and Validation of Esophageal Squamous Cell Carcinoma Risk Prediction Models Based on an Endoscopic Screening Program. *JAMA Netw Open*. 2023;6(1):e2253148. doi:10.1001/jamanetworkopen.2022.53148

29. Jiang L, Xia Z, Zhu R, et al. Diabetes risk prediction model based on community follow-up data using machine learning. *Prev Med Rep*. 2023;35:102358. doi:10.1016/j.pmedr.2023.102358

30. Hu Z, Qiu H, Wang L, Shen M. Network analytics and machine learning for predicting length of stay in elderly patients with chronic diseases at point of admission. *BMC Med Inform Decis Mak*. 2022;22(1):62. doi:10.1186/s12911-022-01802-z

31. Fujihara K, Yamada Harada M, Horikawa C, et al. Machine learning approach to predict body weight in adults. *Front Public Health*. 2023;11:1090146. doi:10.3389/fpubh.2023.1090146

32. Kweon S, Lee JH, Lee Y, Park YR. Personal Health Information Inference Using Machine Learning on RNA Expression Data from Patients With Cancer: Algorithm Validation Study. *J Med Internet Res*. 2020;22(8):e18387. doi:10.2196/18387

33. Chen L, Ashton-James CE, Shi B, et al. Variability in the prevalence of depression among adults with chronic pain: UK Biobank analysis through clinical prediction models. *BMC Med*. 2024;22(1):167. doi:10.1186/s12916-024-03388-x

34. Ren J, Liu D, Li G, Duan J, Dong J, Liu Z. Prediction and Risk Stratification of Cardiovascular Disease in Diabetic Kidney Disease Patients. *Front Cardiovasc Med*. 2022;9:923549. doi:10.3389/fcvm.2022.923549

35. Lee SM, Kim SH, Yoon HJ. Prediction of incident chronic kidney disease in a population with normal renal function and normo-proteinuria. *PLoS One*. 2023;18(5):e0285102. doi:10.1371/journal.pone.0285102

36. King EK, Hsieh MH, Chang DR, et al. Prediction of non-responsiveness to pre-dialysis care program in patients with chronic kidney disease: a retrospective cohort analysis. *Sci Rep*. 2021;11(1):13938. doi:10.1038/s41598-021-93254-0

37. Yu FH, Miao SM, Li CY, et al. Pretreatment ultrasound-based deep learning radiomics model for the early prediction of pathologic response to neoadjuvant chemotherapy in breast cancer. *Eur Radiol*. 2023;33(8):5634-5644. doi:10.1007/s00330-023-09555-7

38. Zhang W, Wan Z, Li X, et al. A population-based study of precision health assessments using multi-omics network-derived biological functional modules. *Cell Rep Med*. 2022;3(12):100847. doi:10.1016/j.xcrm.2022.100847

39. Chandran U, Reps J, Yang R, Vachani A, Maldonado F, Kalsekar I. Machine Learning and Real-World Data to Predict Lung Cancer Risk in Routine Care. *Cancer Epidemiol Biomarkers Prev*. 2023;32(3):337-343. doi:10.1158/1055-9965.EPI-22-0873

40. Song X, Waitman LR, Yu AS, Robbins DC, Hu Y, Liu M. Longitudinal Risk Prediction of Chronic Kidney Disease in Diabetic Patients Using a Temporal-Enhanced Gradient Boosting Machine: Retrospective Cohort Study. *JMIR Med Inform*. 2020;8(1):e15510. doi:10.2196/15510

41. Tavakoli H, Chen W, Sin DD, FitzGerald JM, Sadatsafavi M. Predicting Severe Chronic Obstructive Pulmonary Disease Exacerbations. Developing a Population Surveillance Approach with Administrative Data. *Ann Am Thorac Soc*. 2020;17(9):1069-1076. doi:10.1513/AnnalsATS.202001-070OC

42. Surian NU, Batagov A, Wu A, et al. A digital twin model incorporating generalized metabolic fluxes to identify and predict chronic kidney disease in type 2 diabetes mellitus. *NPJ Digit Med*. 2024;7(1):140. doi:10.1038/s41746-024-01108-6

43. Chen J, Li Y, Liu P, Wu H, Su G. A nomogram to predict the in-hospital mortality of patients with congestive heart failure and chronic kidney disease. *ESC Heart Fail*. 2022;9(5):3167-3176. doi:10.1002/ehf2.14042

44. Shen J, Wang S, Sun H, et al. A novel non-negative Bayesian stacking modeling method for Cancer survival prediction using high-dimensional omics data. *BMC Med Res Methodol*. 2024;24(1):105. doi:10.1186/s12874-024-02232-3

45. Chen S, Shi Y, Hu B, Huang J. A Prediction Model for In-Hospital Mortality of Acute Exacerbations of Chronic Obstructive Pulmonary Disease Patients Based on Red Cell Distribution Width-to-Platelet Ratio. *Int J Chron Obstruct Pulmon Dis*. 2023;18:2079-2091. doi:10.2147/COPD.S418162

46. Sulthan Alikhan J, Alageswaran R, Miruna Joe Amali S. Self-attention convolutional neural network optimized with season optimization algorithm Espoused Chronic Kidney Diseases Diagnosis in Big Data System. *Biomedical Signal Processing and Control*. 2023;85:105011. doi:10.1016/j.bspc.2023.105011

47. Kluckert J, Hötker AM, Da Mutten R, Konukoglu E, Donati OF. AI-based automated evaluation of image quality and protocol tailoring in patients undergoing MRI for suspected prostate cancer. *Eur J Radiol*. 2024;177:111581. doi:10.1016/j.ejrad.2024.111581

48. Vetrano S, Bouma G, Benschop RJ, et al. ImmUniverse Consortium: Multi-omics integrative approach in personalized medicine for immune-mediated inflammatory diseases. *Front Immunol*. 2022;13:1002629. doi:10.3389/fimmu.2022.1002629

49. Kuhlemeier A, Jaki T, Jimenez EY, et al. Individual differences in the effects of the ACTION-PAC intervention: an application of personalized medicine in the prevention and treatment of obesity. *J Behav Med*. 2022;45(2):211-226. doi:10.1007/s10865-021-00274-2

50. Pretzsch CM, Schäfer T, Lombardo MV, et al. Neurobiological Correlates of Change in Adaptive Behavior in Autism. *Am J Psychiatry*. 2022;179(5):336-349. doi:10.1176/appi.ajp.21070711

51. Burger PM, Savarese G, Tromp J, et al. Personalized lifetime prediction of survival and treatment benefit in patients with heart failure with reduced ejection fraction: The LIFE-HF model. *Eur J Heart Fail*. 2023;25(11):1962-1975. doi:10.1002/ejhf.3028

52. Hageman SHJ. The relevance of competing risk adjustment in cardiovascular risk prediction models for clinical practice. doi:10.1093/eurjpc/zwad202

53. Maher CA, Davis CR, Curtis RG, Short CE, Murphy KJ. A Physical Activity and Diet Program Delivered by Artificially Intelligent Virtual Health Coach: Proof-of-Concept Study. *JMIR mHealth and uHealth*. 2020;8(7):e17558. doi:10.2196/17558

54. Sheth A, Yip HY, Shekarpour S. Extending Patient-Chatbot Experience with Internet-of-Things and Background Knowledge: Case Studies with Healthcare Applications. *IEEE Intelligent Systems*. 2019;34(4):24-30. doi:10.1109/MIS.2019.2905748

55. Stephens TN, Joerin A, Rauws M, Werk LN. Feasibility of pediatric obesity and prediabetes treatment support through Tess, the AI behavioral coaching chatbot. *Translational Behavioral Medicine*. 2019;9(3):440-447. doi:10.1093/tbm/ibz043

56. Stein N, Brooks K. A Fully Automated Conversational Artificial Intelligence for Weight Loss: Longitudinal Observational Study Among Overweight and Obese Adults. *JMIR Diabetes*. 2017;2(2):e8590. doi:10.2196/diabetes.8590

57. Barbieri S, Mehta S, Wu B, et al. Predicting cardiovascular risk from national administrative databases using a combined survival analysis and deep learning approach. *Int J Epidemiol*. 2022;51(3):931-944. doi:10.1093/ije/dyab258

58. Xue C, Kowshik SS, Lteif D, et al. AI-based differential diagnosis of dementia etiologies on multimodal data. *Nat Med*. Published online July 4, 2024. doi:10.1038/s41591-024-03118-z

59. Fang Hao, Blair RH. A comparative study: classification vs. user-based collaborative filtering for clinical prediction. *BMC Medical Research Methodology*. 2016;16:1-14. doi:10.1186/s12874-016-0261-9

60. Kosvyra A, Maramis C, Chouvarda I. A data-driven approach to build a predictive model of cancer patients’ disease outcome by utilizing co-expression networks. *Comput Biol Med*. 2020;125:103971. doi:10.1016/j.compbiomed.2020.103971

61. Patro SP, Padhy N, Chiranjevi D. Ambient assisted living predictive model for cardiovascular disease prediction using supervised learning. *Evol Intel*. 2021;14(2):941-969. doi:10.1007/s12065-020-00484-8

62. Kumar D, Balraj K, Seth S, Vashista S, Ramteke M, Rathore AS. An improved machine learning-based prediction framework for early detection of events in heart failure patients using mHealth. *Health Technol*. 2024;14(3):495-512. doi:10.1007/s12553-024-00832-z

63. Liu X, zhou yanju, Zongrun W. Can the development of a patient’s condition be predicted through intelligent inquiry under the e-health business mode? Sequential feature map-based disease risk prediction upon features selected from cognitive diagnosis big data. *International Journal of Information Management*. 2020;50:463-486. doi:10.1016/j.ijinfomgt.2019.05.006

64. Chung K, Park RC. Chatbot-based heathcare service with a knowledge base for cloud computing. *Cluster Computing*. 2019;22(1):1925-1937. doi:10.1007/s10586-018-2334-5

65. Hossain MdIA, Tabassum A, Shamszaman ZU. Deep edge intelligence-based solution for heart failure prediction in ambient assisted living. *Discov Internet Things*. 2023;3(1):11. doi:10.1007/s43926-023-00043-4

66. Feng LH, Lu Y, Ren S, Liang H, Wei L, Jiang J. Development and validation of a dynamic online nomogram for predicting acute kidney injury in cirrhotic patients upon ICU admission. *Front Med*. 2023;10:1055137. doi:10.3389/fmed.2023.1055137

67. Trastulla L, Dolgalev G, Moser S, et al. Distinct genetic liability profiles define clinically relevant patient strata across common diseases. *Nat Commun*. 2024;15(1):5534. doi:10.1038/s41467-024-49338-2

68. Kim SH, Chung K. Emergency situation monitoring service using context motion tracking of chronic disease patients. *Cluster Comput*. 2015;18(2):747-759. doi:10.1007/s10586-015-0440-1

69. L H, Jy L, K S, M K. Estimating heterogeneous survival treatment effects of lung cancer screening approaches: A causal machine learning analysis. *Annals of epidemiology*. 2021;62. doi:10.1016/j.annepidem.2021.06.008

70. Chaix B, Bibault JE, Pienkowski A, et al. When Chatbots Meet Patients: One-Year Prospective Study of Conversations Between Patients With Breast Cancer and a Chatbot. *JMIR Cancer*. 2019;5(1):e12856. doi:10.2196/12856

71. Montagna S, Aguzzi G, Ferretti S, et al. LLM-based Solutions for Healthcare Chatbots: a Comparative Analysis. In: *2024 IEEE International Conference on Pervasive Computing and Communications Workshops and Other Affiliated Events (PerCom Workshops)*. ; 2024:346-351. doi:10.1109/PerComWorkshops59983.2024.10503257

72. Agrawal H, Jain P, Joshi AM. Machine learning models for non-invasive glucose measurement: towards diabetes management in smart healthcare. *Health Technol*. 2022;12(5):955-970. doi:10.1007/s12553-022-00690-7

73. Nghiem N, Atkinson J, Nguyen BP, Tran-Duy A, Wilson N. Predicting high health-cost users among people with cardiovascular disease using machine learning and nationwide linked social administrative datasets. *Health Econ Rev*. 2023;13(1):9. doi:10.1186/s13561-023-00422-1

74. Li J, Chaudhary D, Griessenauer CJ, Carey DJ, Zand R, Abedi V. Predicting mortality among ischemic stroke patients using pathways-derived polygenic risk scores. *Sci Rep*. 2022;12(1):12358. doi:10.1038/s41598-022-16510-x

75. Ye J, Yao L, Shen J, Janarthanam R, Luo Y. Predicting mortality in critically ill patients with diabetes using machine learning and clinical notes. *BMC Medical Informatics & Decision Making*. 2020;20(1):1-7. doi:10.1186/s12911-020-01318-4

76. Murugesh V, Janarthanan P, Kavitha A, Sivakumar N, Jaganathan SCB, Suriyan K. Provisioning a risk predictor model for Alzheimers disease using an improved deep network model. *Multimed Tools Appl*. 2024;83(11):33465-33488. doi:10.1007/s11042-023-16858-w

77. Safa M, Pandian A, Gururaj HL, Ravi V, Krichen M. Real time health care big data analytics model for improved QoS in cardiac disease prediction with IoT devices. *Health Technol*. 2023;13(3):473-483. doi:10.1007/s12553-023-00747-1

78. Mogaveera D, Mathur V, Waghela S. e-Health Monitoring System with Diet and Fitness Recommendation using Machine Learning. In: *2021 6th International Conference on Inventive Computation Technologies (ICICT)*. ; 2021:694-700. doi:10.1109/ICICT50816.2021.9358605

79. Jin J, Kim M, Kim SD. Personalized Health Assistant with Reinforcement Learning. In: *2024 IEEE First International Conference on Artificial Intelligence for Medicine, Health and Care (AIMHC)*. ; 2024:153-156. doi:10.1109/AIMHC59811.2024.00034

80. Silisteanu SC, Covasa M. Reduction of body weight through nutrition intervention reduces chronic low back pain. In: *2015 E-Health and Bioengineering Conference (EHB)*. ; 2015. doi:10.1109/EHB.2015.7391427

81. Gupta A, Basit N. Ranking Actionable Mental Health Advice through a Personalized Video Recommendation System. In: *2022 6th International Conference on Informatics and Computational Sciences (ICICoS)*. ; 2022:140-145. doi:10.1109/ICICoS56336.2022.9930526

82. Kathan A, Harrer M, Küster L, et al. Personalised depression forecasting using mobile sensor data and ecological momentary assessment. *Front Digit Health*. 2022;4:964582. doi:10.3389/fdgth.2022.964582

83. Adams P, Rabbi M, Rahman T, et al. Towards Personal Stress Informatics: Comparing Minimally Invasive Techniques for Measuring Daily Stress in the Wild. In: ; 2014. Accessed October 19, 2024. https://eudl.eu/doi/10.4108/icst.pervasivehealth.2014.254959

84. Chikersal P, Doryab A, Tumminia M, et al. Detecting Depression and Predicting its Onset Using Longitudinal Symptoms Captured by Passive Sensing: A Machine Learning Approach With Robust Feature Selection. *ACM Trans Comput-Hum Interact*. 2021;28(1):1-41. doi:10.1145/3422821

85. Fukazawa Y, Ito T, Okimura T, Yamashita Y, Maeda T, Ota J. Predicting anxiety state using smartphone-based passive sensing. *J Biomed Inform*. 2019;93:103151. doi:10.1016/j.jbi.2019.103151

86. Kanjo E, Kuss DJ, Ang CS. NotiMind: Utilizing Responses to Smart Phone Notifications as Affective Sensors. *IEEE Access*. 2017;5:22023-22035. doi:10.1109/ACCESS.2017.2755661

87. Jacobson NC, Chung YJ. Passive Sensing of Prediction of Moment-To-Moment Depressed Mood among Undergraduates with Clinical Levels of Depression Sample Using Smartphones. *Sensors (Basel)*. 2020;20(12):3572. doi:10.3390/s20123572

88. O’Brien JT, Gallagher P, Stow D, et al. A study of wrist-worn activity measurement as a potential real-world biomarker for late-life depression. *Psychol Med*. 2017;47(1):93-102. doi:10.1017/S0033291716002166

89. Di Matteo D, Fotinos K, Lokuge S, et al. Automated Screening for Social Anxiety, Generalized Anxiety, and Depression From Objective Smartphone-Collected Data: Cross-sectional Study. *J Med Internet Res*. 2021;23(8):e28918. doi:10.2196/28918
