# Supplementary material for: Big Data–Driven Health Portraits for Personalized Management in Noncommunicable Diseases: Scoping Review
Source: J Med Internet Res. 2025 Jun 5;27:e72636. doi: 10.2196/72636 (PMC12179573; doi:10.2196/72636)
Supplement: Multimedia Appendix 7 [file jmir_v27i1e72636_app7.docx]

Figure S1: The scope of big-data-driven health portraits in NCD management.


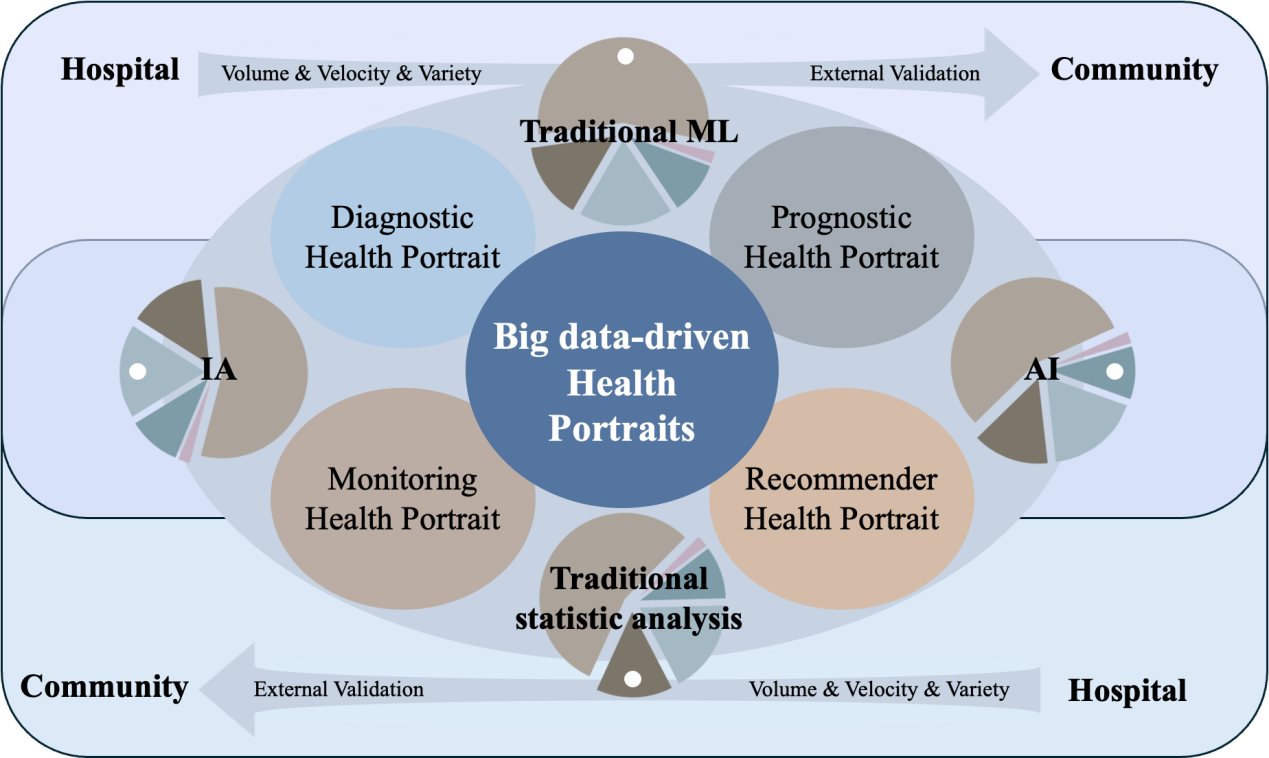


**Fig. S1 The scope of big data-driven health portraits in NCD management.** [ML]: Machine Learning, [IA]: Intelligence Augmented, and [AI]: Artificial Intelligence
