# Supplementary material for: Big Data–Driven Health Portraits for Personalized Management in Noncommunicable Diseases: Scoping Review
Source: J Med Internet Res. 2025 Jun 5;27:e72636. doi: 10.2196/72636 (PMC12179573; doi:10.2196/72636)
Supplement: Multimedia Appendix 8 [file jmir_v27i1e72636_app8.docx]

Figure S2: The roadmap of big data-driven health portraits in NCD management.


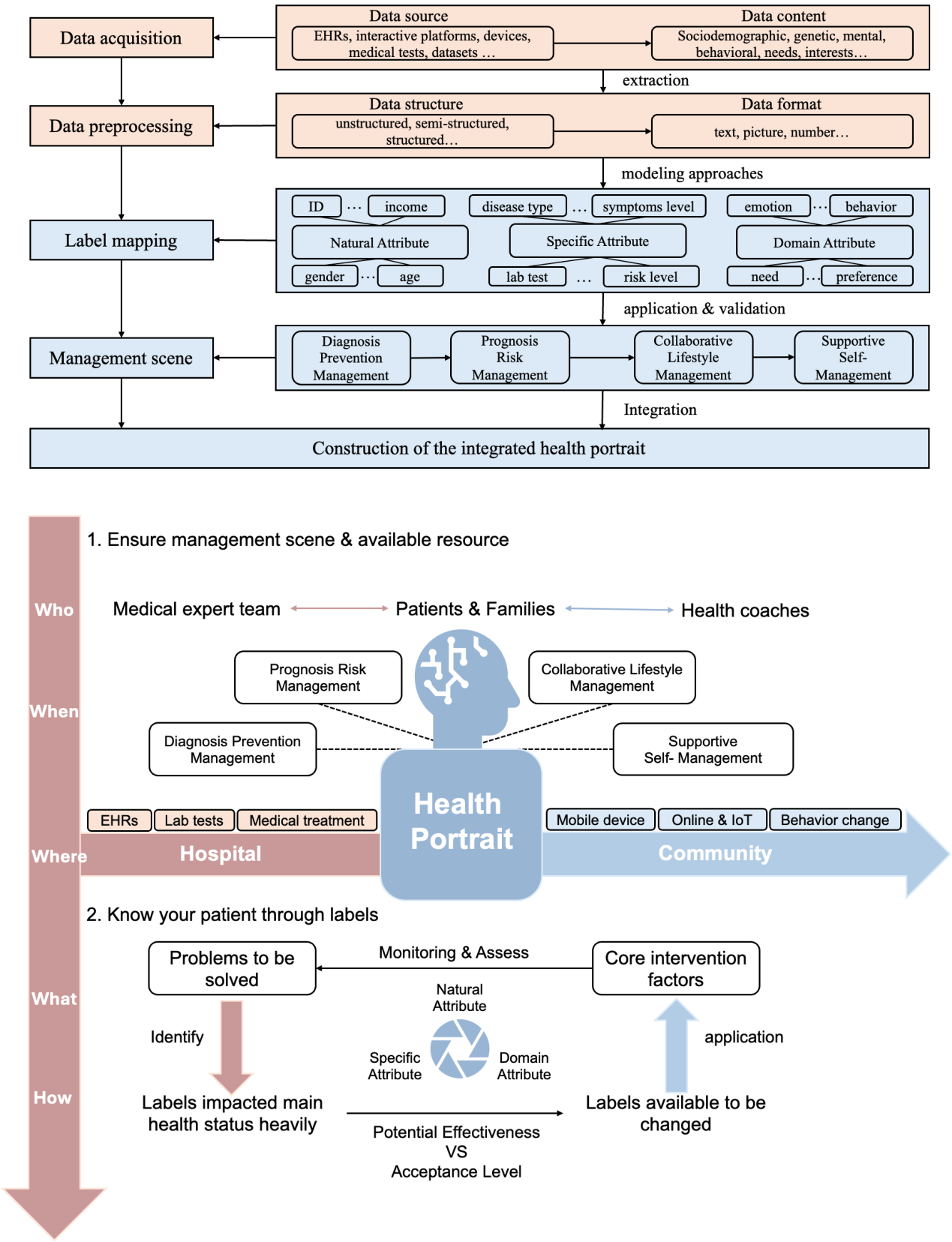


**Fig. S2 The roadmap of big data-driven health portraits in NCD management.** [EHRs]: Electronic Health Records, [Lab]: Laboratory, [IoT]: Internet of Things, [Natural Attribute]: Sociodemographic information, [Domain Attribute]: User information based on knowledge in the health field, mainly including behavioral information and interest information, and [Specific Attribute]: Specific user information extracted according to specific research needs, such as physiological and psychological health attributes
